# Supplementary material for: Asparagine614 Determines the Transport and Function of the Murine Anti-Aging Protein Klotho
Source: Cells. 2024 Oct 21;13(20):1743. doi: 10.3390/cells13201743 (PMC11506777; doi:10.3390/cells13201743)
Supplement: Supplementary file 1 [file cells-13-01743-s001.zip › Figure S1.pdf]

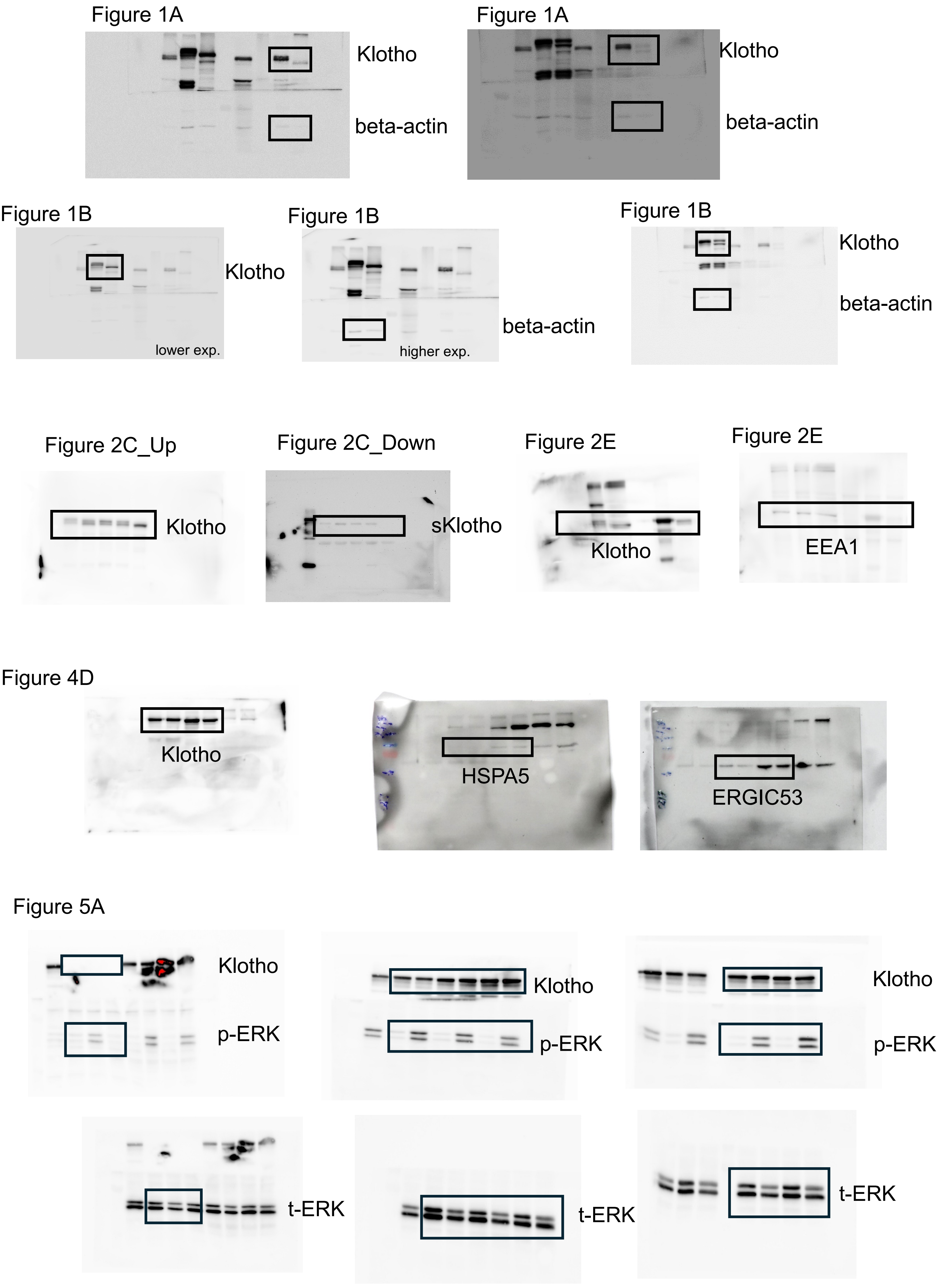

Supplementary Fig. S1: Full-size blots of Western Blots shown in Figs. 1, 2, 4, 5, . Boxed areas mark the parts that were used for the indicated figures.
